# Structural effects on kinetics and a mechanistic investigation of the reaction between DMAD and N–H heterocyclic compound in the presence of triphenylarsine: spectrophotometry approach

**DOI:** 10.1186/s13065-017-0297-x

**Published:** 2017-08-01

**Authors:** Sayyed Mostafa Habibi-Khorassani, Mehdi Shahraki, Mahdieh Darijani

**Affiliations:** Department of Chemistry, Faculty of Science, University of Sistan and Baluchestan, P. O. Box 98135-674, Zahedan, Iran

**Keywords:** Kinetics, Mechanism, Catalyst, N-vinyl heterocyclic

## Abstract

Kinetics and a mechanistic investigation of the reaction between dimethyl acetylenedicarboxcylate (DMAD) and saccharin (**N**–**H** heterocyclic compound) has been spectrally studied in methanol environment in the presence of triphenylarsine (**TPA**) as a catalyst. Previously, in a similar reaction, triphenylphosphine (**TTP**) (instead of triphenylarsine) has been employed as a third reactant (not catalyst) for the generation of an ylide (final product) while, in the present work the titled reaction in the presence of **TPA** leaded to the especial N-vinyl heterocyclic compound with different kinetics and mechanism. The reaction followed second order kinetics. In the kinetic study, activation energy and parameters (Ea, ΔH^‡^, ΔS^‡^ and ΔG^‡^) were determined. Also, the structural effect of the **N**–**H** heterocyclic compound was investigated on the reaction rate. The result showed that reaction rate increases in the presence of isatin (**N**–**H** compound) that participates in the second step (step_2_), compared to saccharin (another **N**–**H** compound). This was a good demonstration for the second step (step_2_) of the reaction that could be considered as the rate- determining step (RDS). As a significant result, not only a change in the structure of the reactant (**TPA instead of TPP**) creates a different product, but also kinetics and the reaction mechanism have been changed.

## Introduction

**Fig. 1:**
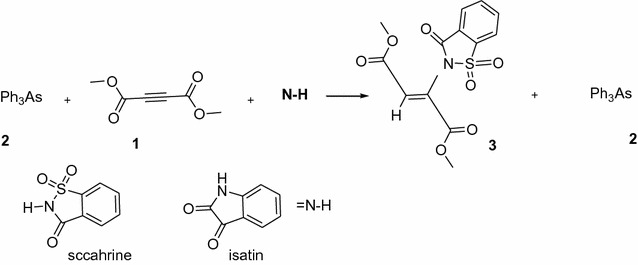
The three-component synthesis of a N-vinylheterocyclic compound [19]

Most compounds that are designated as drugs and are natural have a nitrogen atom. N-vinyl heterocyclic compounds with applications in polymers, natural product analogs, polymeric dyes, pharmaceuticals, etc. are an objective for the organic and medicinal chemist [1–3]. The synthesis of diastereospecific (Z)-N-vinyl compounds previously reported from the reaction between dialkyl acetylenedicarboxylate and **N**–**H** heterocyclic compounds such as saccharin or isatin in the presence of triphenylarsine (**TPA**), (Fig. 1) [4]. **TPA** as an organoarsenic compound is applied in organic synthesis (for example alkene synthesis) [5]. **TPA** with high nucleophilic properties plays the role of catalyst in the titled reaction. Also, the two **N**–**H** heterocyclic compounds that have been used were saccharin and isatin. These heterocyclic compounds and their derivatives have biological and pharmacological effects [6–9]. The similar reactions in the presence of triphenylphosphine (**TPP**) indicated that they have different products [10–12]. The difference between **TPP** and **TPA** is in their nucleophilic properties. Arsonium ylides are more nucleophilic and have more instability than phosphonium ylides [13]. Arsonium ylides react better in the some reactions due to p orbital of carbon has a less overlap with d orbital of adjacent arsenic atom, compared to phosphor atom, thus arsenium ylides are not appeared much more in a form of ylide [14]. Although, kinetics and a mechanistic investigation of some reactions with triphenylphosphine have been reported [15–22], previously. Nevertheless, it has not reported any attempts for similar reactions with triphenylarsine. In this article we report the kinetics of the formation of N-vinyl compound from reaction between dimethyl acetylenedicarboxylate 1 (**DMAD**) and triphenylarsine **2** (**TPA**) with saccharin as a **N**–**H** heterocyclic compound. Synthesis of this reaction has been investigated, previously [4].

### Experimental chemicals and apparatuses used

All acquired chemicals were used without further purification. Dimethyl acetylenedicarboxcylate (**1**), triphenylarsine (**2**) saccharin and isatin as the two **N**–**H** heterocyclic compounds were supplied by Merck (Darmstadt, Germany), Acros (Geel, Belgium) and Fluka (Buchs, Switzerland). Extra pure methanol and ethanol were also obtained from Merck (Darmstadt, Germany). A Cary UV–vis spectrophotometer model Bio-300 with a 10 mm light-path quartz spectrophotometer cell equipped with a thermostated housing cell was used to record the absorption spectra in order to the follow reaction kinetics.

### General procedure

**Fig. 2:**
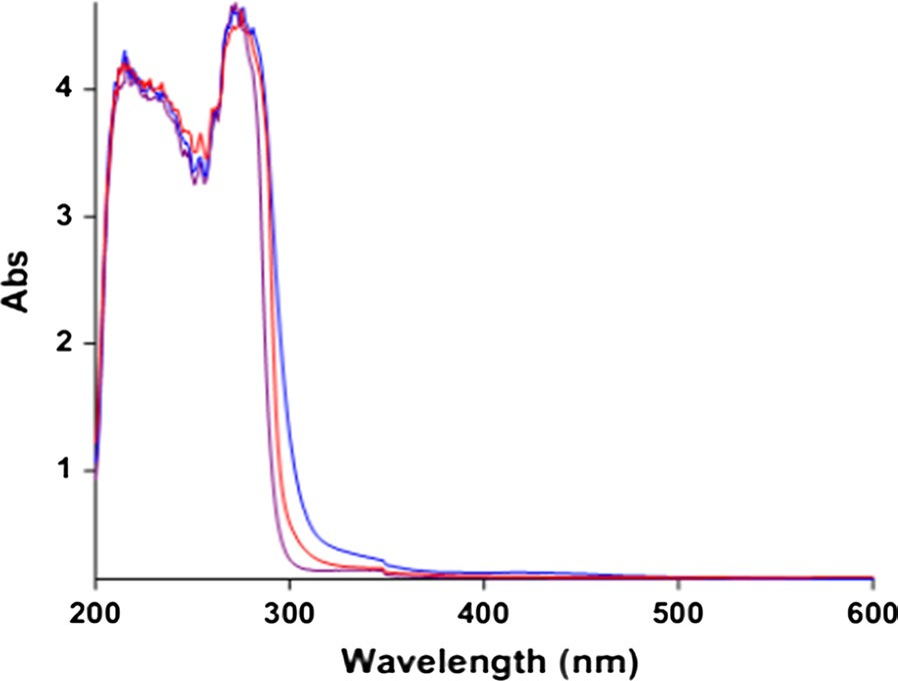
The UV spectrum of 10^−2^M of (**1**), (**N**–**H**) compound and 5 × 10^−3^M of (**2**) as a catalyst in methanol

For the kinetic study of the reaction with a UV spectrophotometer, first it was necessary to find the appropriate wavelength to follow the absorbance change with time. For this purpose 10^−2^ M solution of each reactant containing **(1)** and **N–H** compound and 5 × 10^−3^ M of compound **(2)** were prepared in methanol solvent. The UV–vis spectra of each compound were recorded at 18 °C over a wavelength range of 200–800 nm. Figure 2 shows the spectra of compounds **(1)**, **(2)** and **N**–**H** compound.

**Fig. 3:**
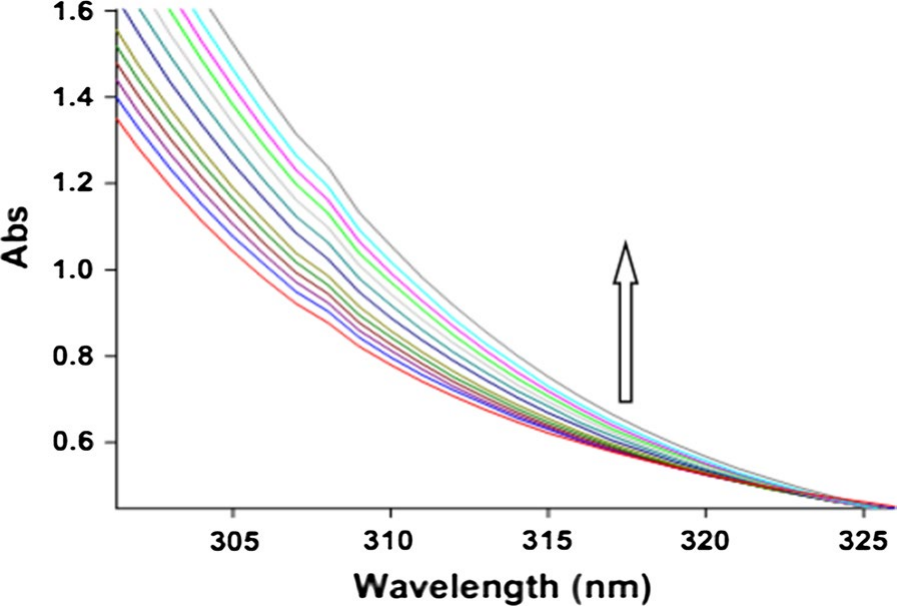
Absorption changes versus wavelengths for the reaction between (**1**) (10^−2^ M), (**2**) (5 × 10^−3^ M) and (2) (10^−2^ M) in methanol for the generation of product 3 at 5 min intervals up to 60 min; the *upward arrow* indicates the direction of the reaction’s progress

In the second experiment, the reaction mixture was started in a 10 mm quartz spectrophotometer cell with mentioned solutions of reactants (**1**), 2 compound and (**2**) with respect to the stoichiometry of each compound in the overall reaction. The absorbance changes of the mixed solution versus wavelengths were recorded until the reaction was finished (Fig. 3).

**Fig. 4:**
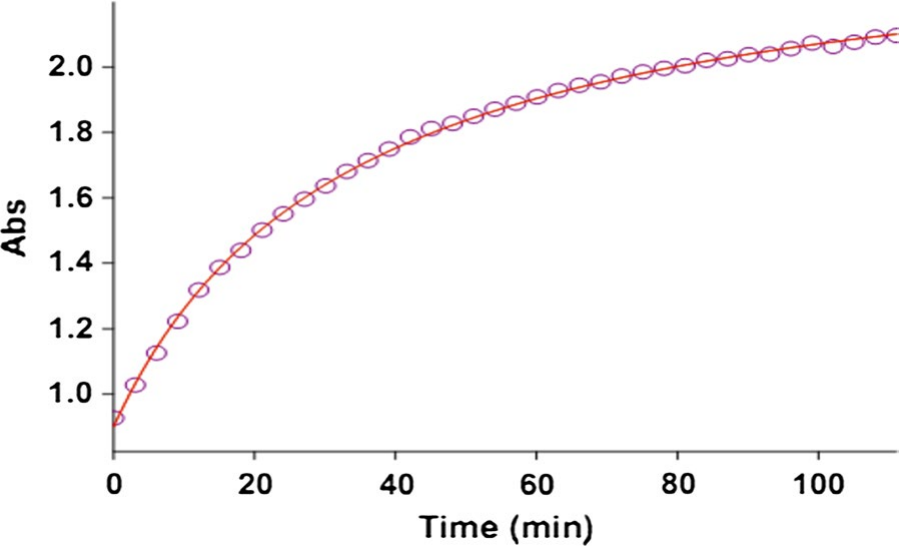
The original experimental absorbance curve versus time at a selected wavelength of 305 nm for the reaction between (**1**) (10^−2^ M), (**2**) (5 × 10^−3^ M catalyst) and (**N**–**H**) (10^−2^ M) in methanol. The *dotted curve* shows experimental values, and the *solid line* is the *fitted curve*

All kinetic measurements were performed by monitoring the absorbance increase at 305 nm because at this wavelength, reactants (**1**), (2), 1 compound have no relatively absorbance values (see Fig. 2). For a linear relationship between absorption and concentration, the UV–vis spectra of compound (**3**) was measured over the concentration range (10^−2^ and 10^−3^ M). In the third experiment, under the same concentration to the previous experiment, we measured the increases of the absorbance of the product with time at an 18 °C temperature and a wavelength of 305 nm (Fig. 4). The second-order rate constant is automatically calculated using the standard equations [23] within the program at 18 °C. In this case, the overall order of rate law can be written as: $a + c = 2$ and the general reaction rate is described by the kinetic following equation:

$${\text{Rate}} = {\text{k}}_{ovr} \left[ { 1\left] {^{\text{a}} } \right[ 2\left] {^{\text{b}} } \right[{\text{N}}{-}{\text{H}}} \right]^{\text{c}}.$$

[2] is catalyst and constant, then, the rate law can be expressed:

1 $${\text{Rate }} = {\text{ k}}_{obs} \left[ 1\right] \, \left[ {{\text{N}} - {\text{H}}} \right]$$

$$\text{k}_{obs} = \text{k}_{ovr} \left[ 2 \right]^{\text{b}}$$

## Results and discussion

In order to determine the partial order with respect to saccharin (**N**–**H** compound) kinetic measurements were performed under pseudo-first-order conditions with twofold excess of DMAD **(1)** by plotting the UV–vis absorbance versus time at a wavelength of 305 nm for the reaction between (**1**) (10^−2^ M), (**2**) (5 × 10^−3^ M) and (**N**–**H**) (5 × 10^−3^M) at 18 °C in methanol.

$${\text{Rate }} = {\text{ k}}_{ovr} \left[ 1\right]^{\text{a}} \left[ 2\right]^{\text{b}} \left[ {{\text{N}} - {\text{H}}} \right]^{\text{c}}$$

$${\text{Rate }} = {\text{ k}}_{obs} \left[ {{\text{N}} - {\text{H}}} \right]^{{\mathbf{c}}} {\text{k}}_{obs} = {\text{ k}}_{ovr} \left[ 1\right]^{\text{a}} \left[ 2\right]^{\text{b}}$$

**Fig. 5:**
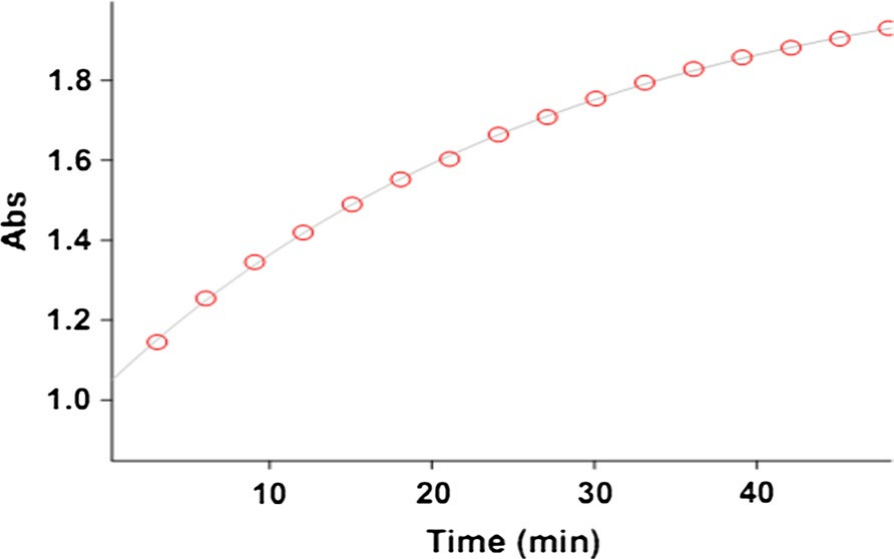
Plot of absorbance versus time at 305 nm for the reaction between (**1**) (10^−2^ M), (**2**) (5 × 10^−3^ M) and **N**–**H** (5 × 10^−3^ M) in methanol. The *dotted curve* shows experimental values, and the *solid line* is the *fitted curve*

The original experimental absorbance versus time data provide a pseudo first order fit curve at 305 nm, which exactly fits the experimental curve (dotted line) Fig. 5. It is obvious that the reaction is of the first order type with respect to saccharin **N**–**H**, $c = 1$. From the second experiment the sum of a and c was obtained two: $+ c = 2$.

From the later experiment, c, is one.

So, order of reaction with respect to** DMAD** (**1**) is one (a = 1).

### Effects of solvents and temperature

The two parameters, dielectric constant and polarity of solvent influence the relative stabilization of the reactants and the corresponding transition state in the solvent environment which in turn effects the rate of the reaction [24, 25]. For examining the effect of the solvent on the rate of reaction, the same kinetic procedure is followed in the presence of ethanol at 18 °C.

The reaction rate is increased in methanol (k_*ovr*_ = 3.0 min^1^ M^−2^) compared to ethanol (k_*ovr*_ = 0.74 min^1^ M^−2^) as the dielectric constant decreased from 32.7 to 24.5 [26], respectively.

### Effect of temperature

The important factor that affects the rate of a chemical reaction is temperature. The influences of temperature on the reaction rate were studied in the range of 18–28 °C with 5 °C intervals for each reaction and the values of second-order rate constants were determined. Table 1 shows kinetic data.

**Table 1:** Reaction rate constants (k_*ovr*_ min^1^ M^−2^) at different temperatures (± 0.1) under the same conditions for the reaction between (**1**) (10^−2^ M), (**2**) (5 × 10^−3^ M^)^ and **N**–**H** compound (10^−2^ M)

λ/nm	Solvent	18 °C ± 0.1	23 °C	28 °C	33 °C
305	Methanol	3.0	3.4	4.0	4.5

The temperature dependency of the rate reaction rate is expressed by the Arrhenius Eq. 2:

2 $$k = Ae^{{\frac{ - Ea}{RT}}}$$

**Fig. 6:**
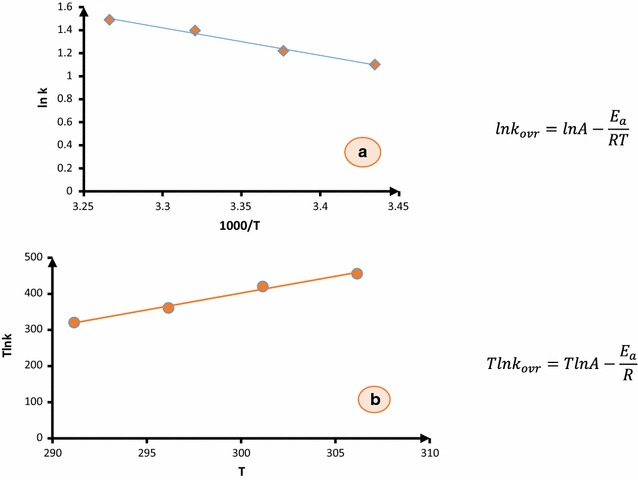
**a** Dependence of second order rate constant (*lnk*_*ovr*_ against *1/T*) on reciprocal temperature for the reaction between reactants (**1**), (**2**) and (**N**–**H**) in methanol measured at wavelength of 305 nm in accordance with the Arrhenius equation for obtaining $\frac{{E_{a} }}{R}$ from the slope. **b** A linearized form of Arrhenius equation (T lnk against T) in order to obtain ln A from the slope

Plotting the graph of *ln k* versus the reciprocal of the temperature (*1/T)* yields a straight line with a slope of *E*_*a*_ */R* and an intercept of *ln A* (Fig. 6).

On the basis of Eyring Eq. 3 [27] and linearized form of the Eyring Eq. 4 [28]:

3 $$ln\frac{k}{T} = - \frac{\Delta H\ddag }{RT} + \frac{{\Delta S^{\ddag } }}{R} + ln\frac{{k_{B} }}{h}$$

4 $${\text{T ln}}\frac{\text{k}}{\text{T}} = \left( { - \frac{{\Delta {\text{H}}^{\ddag } }}{\text{R}}} \right) + {\text{T}}\left( {\frac{{\Delta {\text{S}}^{\ddag } }}{\text{R}} + { \ln }\frac{{{\text{k}}_{\text{B}} }}{\text{h}}} \right)$$

**Fig. 7:**
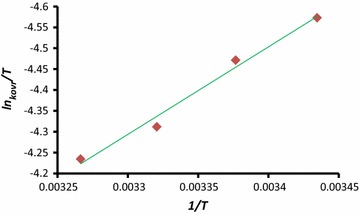
Eyring plot (*ln k*_*ovr*_ */T* versus *1/T*) according to Eq.5 for the reaction between (**1**), (**2**) and (**N**–**H**) compounds in the methanol

Plotting the graph of *ln k/T* versus the reciprocal of the temperature *1/T* and also *T lnk/T* against *T* yields a straight lines, from which, the values for ∆H^‡^ (activation enthalpy), ∆S^‡^ (activation entropy) can be determined (see Fig. 7; Table 2).

**Table 2:** Activation parameters (∆S^‡^, ∆H^‡^, ∆G^‡^ and ln A) at 18 °C for the reaction between (**1**), (**2**) and **N**–**H** compounds

	∆H^‡^ kJ mol^−1^	∆S^‡^ kJ mol^−1^K^−1^	T∆ S^‡^ kJ mol^−1^	E_a_^a^ kJ mol^−1^	E_a_^b^kJ mol^−1^	A M^−1^min^−1^
Arrhenius Eq. 2. and Eyring Eq. 3	17.4 ± 0.5	−175.8 ± 1.7	−51.17	19.9 ± 0.5	19.8 ± 0.5	1.1 × 10^4^

∆G^‡^ = 68.59 ± 1.02 at 18 °C

^a^From Arrhenius Eq. 2

^b^From equation E_a_ = ∆H^‡^ +RT

The Gibbs activation energy has been evaluated from the following form of the Gibbs–Helmholtz Eq. 5:

5 $$\Delta {\text{G}}^{\ddag } = \Delta {\text{H}}^{\ddag } - {\text{T}}\Delta {\text{S}}^{\ddag }$$

The Gibbs activation energy is essentially the energy requirement for a molecule (or a mole of them) to undergo the reaction. It is of interest to note that the Gibbs activation energy is positive. The Gibbs activation energy changed with enthalpy and entropy. Sometimes ∆H^‡^ is the main provider, and sometimes T∆S^‡^ consider the main provider in Eq. 5 that refer to enthalpy or entropy-controlled reaction, respectively.

As can be seen from the Table 2, T∆S^‡^ (51.17 kJ mol^−1^K^−1^) is much greater than ∆H^‡^ (17.5 kJ mol^−1^) which implies that the reaction is entropy-controlled.

### Effect of N–H compounds

**Fig. 8:**
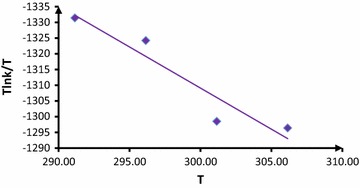
A linearized form of Eyring Eq. 4 [Tlnk_*ovr*_/T against T] for the reaction between (**1**), (**2**) and (**N**–**H**) compounds in the methanol

This section focuses exclusively on the effects of the different structural of N–H compounds on the reaction rate for generation of a N-vinyl heterocyclic compound. A plot of absorbance vs. time, is shown in Fig. 8 for the reaction with isatin as another **N**–**H** heterocyclic compound under same condition with previous experiment.

The rate of reaction speeds up in comparison with saccharin. This experiment indicated that **N**–**H** compounds (saccharin or isatin) participated in the rate-determining step (RDS) of the reaction mechanism (step_2_).

### Mechanism

On the basis of experimental results and reports on literatures [4] a speculative mechanism is represented in Fig. 10.

**Fig. 9:**
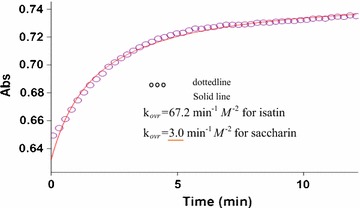
The original experimental absorbance curve versus time at a selected wavelength of 305 nm for the reaction between **(1)** (10^−2^ M), (**2**) (5 × 10^−3^ M) and isatin (**N**–**H**) (10^−2^ M) in methanol. The *dotted curve* shows experimental values, and the *solid line* is the *fitted curve* at 18 °C

**Fig. 10:**
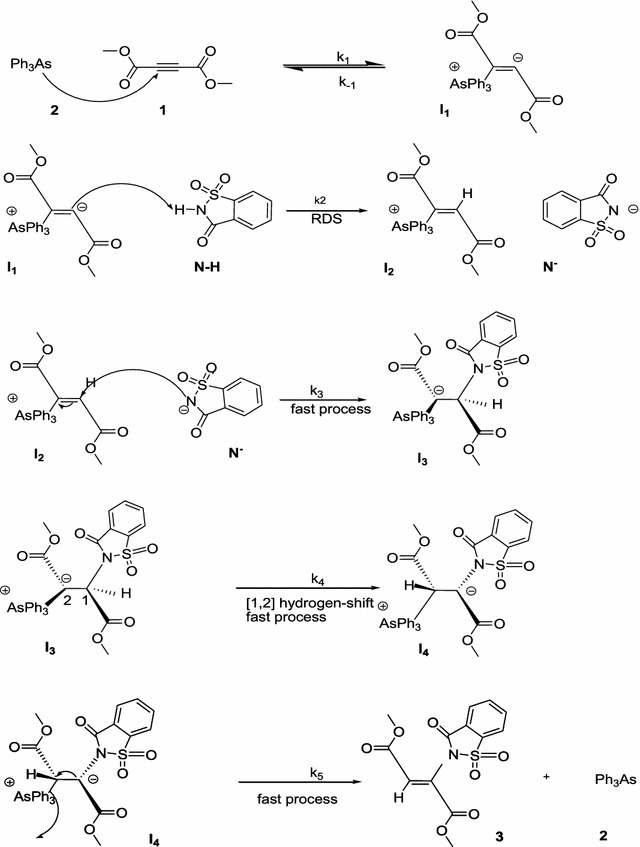
Speculative mechanism for the reaction between (**1**) and (**N**–**H**) compound (saccharin)in the presence of a catalyst (**2**) for generation of N-vinyl heterocyclic compound **3** in methanol

To investigate which step of the reaction mechanism is a rate determining step (RDS), further experiments were performed as follows:

A series of experiments, containing two-component reactions between dimethyl acetylenedicarboxylate (**DMAD**) (**1**) and triphenylarsine (**TPA**) (**2**) (Re. 1), dimethyl acetylenedicarboxylate (**1**) and **N**–**H** compound (Re. 2), and then** N**–**H** compound and triphenylarsine (**2**) (Re. 3) were carried out under the same concentration of each reactant (10^−2^ M) at 18 °C. Both reactions (Re. 2 and Re. 3) had no progresses, in fact, there were no reactions between **N**–**H** compound (isatin or saccharin) and (**2**) or (**1**) due to the lack of progress. The Re. 1 was monitored by recording scans of the entire spectra with 5 min intervals reaction time (5 min) at 18° C (Fig. 9). According to these observations, starting reaction between reactants (**1**) and (**2**) is the more rapidly occurring reaction amongst competing reactions (see step_1_, Fig. 10).

This step (k_1_) containing the reaction between (**1**) and (**2**) (k_1_ = 6.18 min^−1^ M^−2^) is faster than the overall reaction (k_*ovr*_ = 3.0 min^−1^ M^−2^) between (**1**), (**2**) and** N**–**H** heterocyclic compound. Hence, step_1_ could not be a RDS. Step_3_ (k_3_) is an intramolecular reaction between two ionic species (**I**_**2**_ and **N** ^**−**^) which is inherently fast in a liquid phase (methanol) [29–31]. Step_4_ (k_4_) is also fast because of [1, 2] hydrogen-shift process (**I**_**3**_). In addition, step_5_ (k_5_) is an intermolecular reaction between the two parts of a dipole component (I_4_) which is a rapid reaction. Perhaps, step_2_ (k_2_) is a rate determining step. In order to check this possibility, the rate law is written using the final step of the proposed mechanism in Fig. 10 for the generation of product **3**:

6 $$Rate = k_{5} \left[ {I_{4} } \right]$$

By applying the steady state assumption in obtaining the concentration of intermediates (**I**_**4**_, **I**_**3**_, **I**_**2**_ and **I**_**1**_) the calculated overall rate law equation is:

7 $$Rate = \frac{{k_{2} k_{1} \left[ 1 \right]\left[ 2 \right]\left[ {N - H} \right]}}{{k_{ - 1} + k_{2} \left[ {N - H} \right]}} _{ }$$

Equation 7 doesn’t involve *k*_3_, *k*_*4*_ and *k*_*5*_, hence steps 3, 4 and 5 cannot be the rate determining step, nevertheless the rate law contains *k*_*1*_ and *k*_*2*_, and therefore, there is two possibilities for the rate determining step. If *k*_*2*_ is a rate determining step, the speculation that $k_{ - 1} \gg k_{2} \left[ {N - H} \right]$ is logical, and thus the rate law can be stated as:

$$Rate = \frac{{k_{2} k_{1} \left[ 1 \right]\left[ 2 \right]\left[ {N - H} \right]}}{{k_{ - 1} }}$$

$$k_{obs} = \frac{{k_{2} k_{1} \left[ 2 \right]}}{{k_{ - 1} }}$$

Due to compound **(2)** is a catalyst, its concentration is constant, and so the rate law can be stated:

8 $$Rate = k_{obs} [1][N - H]$$

This Eq. 8 is compatible with the second-order experimental rate law (Eq. 1) which means that step_2_ (k_2_) is the RDS.

Another possibility is considered for step_1_ (*k*_*1*_) as a rate determining step, in this case, it is reasonable to accept this assumption, $k_{ - 1} \ll k_{2} \left[ {N - H} \right]_{2}$, under this condition the rate law can be written as:${\text{Rate}} = \frac{{{\text{k}}_{2} {\text{k}}_{1} \left[ 1 \right]\left[ 2 \right]\left[ {{\text{N}} - {\text{H}}} \right]}}{{{\text{k}}_{2} \left[ {{\text{N}} - {\text{H}}} \right]}} _{ }$ and then,

$${\text{Rate}} = {\text{k}}_{1} \left[ 1 \right]\left[ 2 \right]$$

Compound (2**)** is a catalyst and its concentration is constant, so the rate law can be expressed:

$$k_{obs} = k_{1} \left[ 2 \right]$$

9 $$Rate = k_{obs} \left[ 1 \right]$$.

Equation 9 is a rate law for the first-order kinetic reaction that is not agreement with the experiment results (Eq. 1). The acceptable rate law, Eq. 8, involving **N**–**H** compound and compound **(1)** is a rate determining step which depends on the concentration of **N**–**H** compound. In previous section, can be seen that the different structures of **N**–**H** compound (containing saccharin or isatin) with their different ability of acidity and geometries had a great effect on step_2_ (k_2_).

**Fig. 11:**
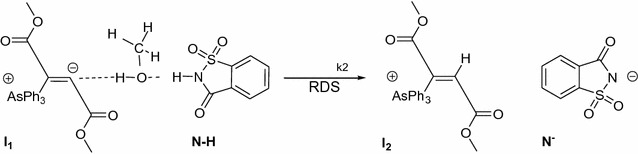
Comparison between the steric effect of CH_3_OH or C_2_H_5_OH on a proton transfer process between the **N**–**H** and **I**_**1**_

Although, **I**_**1**_ (intermediate) in step_2_ can be stabilized easily by dipole–dipole interactions in the presence of solvent with higher dielectric constant which reduces the reaction rate. Nevertheless, a proton from **N**–**H** compound can be transferred easily towards intermediate **I**_**1**_ (see Fig. 11), in the presence of a less hindrance solvent such as methanol, compared to ethanol. This phenomenon increases the rate of reaction. It seems that less steric effect of solvent such as methanol in step_2_ of the reaction has a more effect on enhancement of reaction rate, compared to its dielectric constant that can be stabilized more **I**_**1**_ species and subsequently reduces the reaction rate. For the present work, the reaction rate in the presence of methanol is 4.5 times more than ethanol.

## Conclusions

1. Kinetics for the formation of the N-vinyl heterocyclic compounds was examined in the presence of triphenylarsine (**TPA**) as a catalyst, (**DMAD**) and** N**–**H** heterocyclic compound in methanol using UV–vis spectrophotometer technique. The results demonstrated that the overall order of the reaction is two and the partial orders with regard to each reactant (**1**) or **N**–**H** heterocyclic compound is one.
2. Previously, in a similar reaction, with triphenylphosphine (**TPP**) (instead of triphenylarsine (**TPA**) in the current work), the generated product was an ylide, while in this work is a N-vinyl heterocyclic compound.
3. Different behavior of both reactants (**TPP** or** TPA**) provides a different mechanism and kinetics for both the previous or present works.
4. In the previous work, the reaction followed second-order kinetics and step_1_ of reaction was recognized as a rate determining step. The rate law depended on concentration of (**DMAD**) and (**TPP**) and was independent of concentration of **N**–**H** heterocyclic compound, while in present work, step_2_ of the reaction is a rate determining step (RDS) and the rate law depends on concentrations of both (**DMAD**) and **N**–**H** heterocyclic compound. Herein (**TPA**) has a catalyst role in the reaction medium.
5. In the present work, the structural effect of **N**–**H** heterocyclic compound on the reaction rate was investigated in the presences of isatin as another N–H compound that participates in the second step (step_2_), compared to saccharin. This is a good demonstration for the second step of the reaction (step_2_) that could be considered the RDS.
6. Reaction rate is accelerated by increasing the temperature and the dielectric constant of solvent.
7. Also, enhancement of the steric effect on the structure of solvent from methanol to ethanol can be considered as an effective factor for a proton transfer process between **N**–**H** heterocyclic compound and intermediate **I**_**1**_. Less hindrance in methanol has a great effect on enhancement of the reaction rate, compared to ethanol.
8. The reaction is entropy-controlled (T∆S^‡^ is much greater than ∆H^‡^).
